# Supplementary material for: Codesigning an intervention to strengthen COVID-19 vaccine uptake in Congolese migrants in the UK (LISOLO MALAMU): a participatory qualitative study protocol
Source: BMJ Open. 2023 Jan 13;13(1):e063462. doi: 10.1136/bmjopen-2022-063462 (PMC9842599; doi:10.1136/bmjopen-2022-063462)
Supplement: Author's manuscript [file bmjopen-2022-063462.draft_revisions.pdf]

BMJ Open is committed to open peer review. As part of this commitment we make the peer review history of every article we publish publicly available.

When an article is published we post the peer reviewers' comments and the authors' responses online. We also post the versions of the paper that were used during peer review. These are the versions that the peer review comments apply to.

The versions of the paper that follow are the versions that were submitted during the peer review process. They are not the versions of record or the final published versions. They should not be cited or distributed as the published version of this manuscript.

BMJ Open is an open access journal and the full, final, typeset and author-corrected version of record of the manuscript is available on our site with no access controls, subscription charges or pay-per-view fees (http://bmjopen.bmj.com).

If you have any questions on BMJ Open's open peer review process please email info.bmjopen@bmj.com

BMJ Open

Co-designing an intervention to strengthen vaccine uptake in Congolese migrants in the UK (LISOLO MALAMU): a participatory study protocol

Journal:	*BMJ Open*
Manuscript ID	bmjopen-2022-063462
Article Type:	Protocol
Date Submitted by the Author:	01-Apr-2022

Complete List of Authors:	Crawshaw, Alison; St George's University of London, Institute for Infection and Immunity<br>Hickey, Caroline; Hackney Council for Voluntary Service, Hackney Refugee and Migrant Forum<br>Lutumba, Laura Muzinga; Hackney Congolese Women Support Group<br>Nkembi, Sarah; Hackney Congolese Women Support Group<br>Kitoko, Lusau Mimi; Hackney Congolese Women Support Group<br>Knights, Felicity; St George's Hospital Medical School<br>Ciftci, Yusuf; Doctors of the World UK<br>Goldsmith, Lucy; St George's University of London, Infection and Immunity Research Institute, and Population Health Research Institute<br>Vandrevala, Tushna; Kingston University, Centre for Applied Health and Social Care Research, Faculty of Health, Social Care and Education; Kingston University and St George's University of London, Centre for Applied Health and Social Care Research, Faculty of Health, Social Care and Education,<br>Forster, Alice S; Our Future Health<br>Hargreaves, Sally; St George's University of London, Institute for Infection and Immunity

Keywords:	PUBLIC HEALTH, QUALITATIVE RESEARCH, PRIMARY CARE

SCHOLARONE™
Manuscripts

Title: Co-designing an intervention to strengthen vaccine uptake in Congolese migrants in the UK (LISOLO MALAMU): a participatory study protocol

Authors

Alison F Crawshaw acrawsha@sgul.ac.uk

Caroline Hickey caroline@hcvs.org.uk

Laura Muzinga Lutumba hcwsg@outlook.com

Lusau Mimi Kitoko hcwsg@outlook.com

Sarah Luti Nkembi hcwsg@outlook.com

Felicity Knights fknights@sgul.ac.uk

Yusuf Ciftci yusufciftci4@gmail.com

Lucy P Goldsmith lgoldsmi@sgul.ac.uk

Tushna Vandrevala t.vandrevala@sgul.kingston.ac.uk

Alice S Forster alice.forman@ourfuturehealth.org.uk

Sally Hargreaves s.hargreaves@sgul.ac.uk

Corresponding author

Sally Hargreaves

s.hargreaves@sgul.ac.uk

The Migrant Health Research Group, Institute for Infection and Immunity, St George's, University of London, Cranmer Terrace, London, SW17 0RE, UK

Author affiliations

The Migrant Health Research Group, Institute for Infection and Immunity, St George's, University of London, Cranmer Terrace, London, SW17 0RE, UK (AF Crawshaw, F Knights, LP Goldsmith, S Hargreaves)

Centre for Applied Health and Social Care Research, Faculty of Health, Social Care and Education, Kingston University and St George's, University of London, UK (T Vandrevala)

Hackney CVS, The Adiaha Antigha Centre, 24-30 Dalston Lane, London, E8 3AZ (C Hickey)

Hackney Congolese Women Support Group, c/o Hackney CVS, The Adiaha Antigha Centre, 24-30 Dalston Lane, London, E8 3AZ (LM Lutumba, LM Kitoko, SL Nkembi)

For peer review only

Doctors of the World UK, London, E14 5AA, UK (Y Ciftci)

Our Future Health, New Bailey, 4 Stanley Street, Manchester, M3 5JL, UK (AS Forster)

For peer review only

Abstract

Introduction

Adult migrants are at risk of under-immunisation and are likely to need catch-up vaccination to bring them in line with the UK schedule. The COVID-19 pandemic has highlighted and exacerbated inequities in vaccine uptake, with migrants facing additional barriers to information, low vaccine confidence, and access to vaccine services. There is a need for participatory and theory-based research that meaningfully engages underserved migrant groups to make sense of their experiences and beliefs about vaccination and uses these insights to co-produce tailored interventions which can increase uptake. COVID-19 vaccination provides a unique entry-point and opportunity to explore these issues in tandem with addressing routine immunisation gaps and developing more culturally-sensitive routine vaccination services.

Methods and analysis

LISOLO MALAMU ('Good Talk') is a community-based participatory research study which uses co-design, design thinking and behaviour change theory to engage adult Congolese migrants in developing a tailored intervention to increase vaccine uptake. A community-academic coalition will lead and co-design the study. The study will involve i) in-depth interviews with adult Congolese migrants (foreign-born, >18 years), ii-iii) interviews and consensus workshops with clinical, public health and community stakeholders, and iv) co-design workshops with adult Congolese migrants. Qualitative data will be analysed iteratively, using Thematic Analysis, and mapped to the Theoretical Domains Framework, with participation from the coalition in discussing and interpreting findings and selecting intervention functions to guide the co-design workshops. Sociodemographic data of interview participants will be summarised using descriptive statistics. The study will run from approximately November 2021-November 2022.

Ethics and dissemination

Ethics approval has been granted by the St George's University Research Ethics Committee (REC reference 2021.0128). Study findings will be widely disseminated by the coalition through local community organisations in Hackney and broader academic and policy stakeholders, including a final celebration event. Recommendations for a future larger scale study and testing of prototyped interventions will be made.

Strengths and limitations of this study:

For peer review only

Strengths

- This study will directly respond to ongoing calls for community-centred and participatory approaches to engaging migrants in routine and COVID-19 vaccination, by implementing a value-driven and reciprocal approach to conducting a study addressing the needs of an underserved community.
- The target population was selected following a comprehensive systematic review of the evidence (1) and pre-engagement scoping work conducted with migrant community representatives in London, UK. (2, 3)
- It aims to co-produce a tailored intervention to address specific barriers to, and strengthen, vaccine uptake for COVID-19 and routine vaccines in adult Congolese migrants (including MMR, Td/IPV, and HPV) as set out by UKHSA guidance (4), and has been co-designed with, and will be co-delivered by, a coalition formed of academic researchers, a council for voluntary service (a local charity which offers services and support for local voluntary and community organisations), and a Congolese community-based organisation.

Limitations

- As this study is tailored to the Congolese migrant population, other migrants who also face barriers to vaccine uptake are not included. Whilst we can draw some conclusions about the experiences of other Black migrants who face similar historical and cultural barriers to uptake of routine and COVID-19 vaccines, our ability to generalise the findings to all migrant communities might be limited.
- Co-designed intervention prototypes will not be formally implemented and evaluated in this study, however recommendations will be made so that this can be done in a future phase.

Keywords: community-based participatory research; transients and migrants; health disparities; vaccination uptake; community-academic research partnerships; co-production; intervention design

For peer review only

Introduction

Adult migrants (foreign-born individuals) in Europe, particularly those from low- and middle-income countries, are at risk of under-immunisation for routine vaccinations (5-8) and have been involved in outbreaks of serious vaccine-preventable diseases, including measles (9). The reasons for their undervaccination are multiple and complex, and include differing vaccination schedules in migrants' home countries, practical factors relating to access and availability, historical and cultural reasons, and other individual and social processes, which can occur before, during, and after migration (1, 10, 11).

Unlike children, who are typically aligned with the host country's vaccination schedule upon attending school, adult and adolescent migrants are not routinely incorporated into vaccination programmes on arrival to most European countries, including the UK (12), due to lack of guidance, and well-documented barriers to accessing health systems (13). Our recent systematic review (1) confirmed that access barriers including language, literacy, communication, practical, legal, and service barriers, are particularly important barriers to vaccination for migrants in transit and host countries, and that specific factors including country of origin (particularly African), having more recently migrated, and being an asylum seeker or refugee, could be determinants of under-immunisation in migrants.

The World Health Organization has launched its new Immunization Agenda 2030 (IA2030) (14) with an emphasis on achieving equitable access to vaccination for vulnerable populations and integrating vaccination throughout the lifecourse, including catching-up adolescent and adult migrants with missed vaccines, doses and boosters, to close immunisation gaps. WHO and ECDC guidance for catch-up vaccination is available (15, 16) and in the UK specific guidance on the 'vaccination of individuals with uncertain or incomplete immunisation status' is available from UKHSA (17), although the implementation of this guidance in practice is unknown and thought to be inconsistent, with low awareness of the guidance by clinicians in primary care (18).

Besides routine vaccination, there have been striking disparities in COVID-19 vaccine uptake, with UK and US evidence showing migrants and minoritised populations – particularly Black groups – having some of the lowest uptake rates to date. (19-21) The COVID-19 pandemic has exposed inequities in engaging adult migrants and other under-served populations in vaccination programmes (11, 22) and highlighted how structural racism and marginalisation serve to perpetuate their poorer health outcomes (23). If not adequately addressed, the enduringly low levels of COVID-

For peer review only

19 vaccine uptake in these populations are likely to widen existing inequities. A lack of adult immunisation programmes worldwide – and therefore crucial infrastructure for delivering routine and COVID-19 vaccinations globally – also poses a barrier to achieving equitable COVID-19 vaccine deployment and uptake among adults, and must be addressed (24).

Emerging evidence, including from our pre-engagement work done to inform this study (reported elsewhere (2, 3)) attributes lower COVID-19 vaccine uptake rates in minoritised and migrant populations to a lack of confidence – specifically due to the spread of misinformation and conspiracy theories, mistrust in the medical establishment and government, and concerns about side effects – and access barriers, including physical access, language and communication barriers (9, 25-30). In the UK, community-research partnerships have been mobilised to engage with ethnic communities and address the disparities in COVID-19 vaccine uptake including through outreach and the development of culturally relevant health information and messages, (31, 32) and public bodies and charities have now translated official information and guidance into multiple languages and developed toolkits (33-35). However, there are fewer initiatives to use community partners as equal partners in research and community-based participatory research (CBPR) studies specifically engaging and involving migrant populations around vaccination, and indeed on other health topics. (36) To date, few studies have closely and carefully explored the barriers and facilitators in specific migrant sub-populations with inequitable uptake, and for whom mainstream interventions and resources have failed to reach or influence, with the purpose of using these insights to co-produce tailored interventions. This is amid calls from the World Health Organization (WHO) and the European Centre for Disease Prevention and Control (ECDC) for more participatory research on the determinants of health in migrant populations, and better understanding of the needs and perspectives of refugees and migrants in vaccination initiatives (37, 38). There is a real opportunity for the renewed focus on engaging underserved populations brought about by COVID-19 to be carried over to routine vaccinations, with improved inclusion of migrant populations, who continue to be overlooked.

CBPR approaches, which aim to equitably involve all partners in the research process (39), hold potential to tackle this complex issue because they recognise the value of lived experience and emphasise the sharing of power, ensuring research is embedded within, conducted in collaboration with, and tailored to, a specific community (40, 41). Behaviour change models and frameworks, such as the Theoretical Domains Framework, can be used in tandem to understand the context of behaviour and design targeted interventions (42), and have been applied in a variety of health settings, including vaccination (43). The relevance of CBPR approaches to migrant health research

For peer review only - http://bmjopen.bmj.com/site/about/guidelines.xhtml

has been noted (44) and evidence shows that interventions driven by insights from the communities they are designed to serve are more cost-effective and lead to better results for health behaviour outcomes than traditional interventions (45, 46). A systematic review which looked at strategies for addressing vaccine hesitancy globally found that multi-component and dialogue-based interventions were most effective, and recommended that strategies should be carefully tailored to the target population, their specific reasons for hesitancy, and context (47). Recent research around COVID-19 testing with people of Black ethnicity in the UK highlighted the role of mistrust, alienation and stigmatisation in creating barriers to testing for this population, and recommended that health communications address these issues and build trust through local credible sources (48). Evidence also supports the importance of locality in establishing and maintaining trust and credibility in the processes of community engagement and health promotion, and for developing contextually-specific, tailored interventions (48).

To inform this study, we conducted a systematic review of the literature on migrant vaccine uptake in Europe (1) (including local grey literature) and 3 online pre-engagement workshops with migrant community representatives in London (in December 2020-February 2021), to scope out community perceptions towards COVID-19 vaccination and barriers and facilitators to uptake, and found support within communities to participate in research to co-develop solutions (2, 3). Based on our key findings that recent migrants, refugees and asylum seekers, and those from Africa may be at risk of being under-immunised (1), local data showing that people from the Black community in Hackney have some of the lowest rates of vaccine coverage for routine childhood vaccination and COVID-19 vaccination (49, 50), evidence of widespread access and confidence barriers affecting migrants' COVID-19 vaccine uptake locally, and the relationships built with community organisations during our pre-engagement work, a community-academic partnership was formed and adult Congolese migrants (predominantly from the Democratic Republic of Congo, DRC) were chosen as our target population. The DRC migrant population in the UK has a large proportion of older adult migrants who began migrating to the UK in the late 1980s, many as political refugees, with increased flows since the late 1990s (51). DRC refugees were the fourth most common nationality to be resettled to the UK through the UK's four main refugee resettlement schemes between 2015-2020 (52) (n=1774) and recent unpublished data suggest that UK-bound adult DRC refugees may be significantly under-immunised compared to the UK immunisation schedule (53). The Congolese population in the UK is historically underserved and there is very limited literature on their health-seeking behaviour and health outcomes. This study will therefore make an important contribution to the evidence base.

For peer review only

The aim of this study is to use CBPR approaches to engage and involve Congolese migrants in Hackney in the co-design of a tailored intervention to increase vaccine uptake. It seeks to i) gather information about and make sense of Congolese adult migrants' beliefs, experiences of vaccination (routine, COVID-19, catch-up), access to healthcare, and other lived experiences with respect to vaccination, ii) understand local pathways, processes and services, and considerations for implementation of interventions with key stakeholders; and iii) co-design a tailored intervention to strengthen vaccine uptake with Congolese migrants, which can be formally evaluated.

Methods and analysis

Study design

LISOLO MALAMU (Lingala for 'Good Talk') is a CBPR study which uses co-design methods, the principles of design thinking (an iterative, solutions-based approach to problem-solving that starts with the needs and desires of the target population) (54) and behaviour change theory (42) to engage Congolese migrants in developing a tailored intervention to increase vaccine uptake. The comprehensive and practical Theoretical Domains Framework (TDF) and accompanying Behaviour Change Wheel (BCW), specifically developed for implementation research, were chosen to guide the intervention design in this study (42). The study name was decided in consultation with the community to reflect the project's ethos of providing a platform for meaningful conversations around vaccination. It involves 4 main activities: 1) community days, involving qualitative in-depth interviews with Congolese migrants, which began in January 2022, 2) in-depth interviews (IDIs) with local clinical, public health and community stakeholders, 3) consensus workshops with the same key stakeholders, and 4) co-design workshops with Congolese migrants. An evaluation component will be embedded across all activities. The study process is illustrated in Figure 1. Good practices, challenges and facilitators relating to the implementation of the study and the method of using co-design will also be documented. An academic-community research partnership has been formed (referred to herewith as "the coalition") to co-design, steer and conduct the study.

[FIGURE 1]

Setting and population

The study is being carried out in Hackney, London, UK, a highly diverse London borough, in which more than 89 languages are spoken and around 40% of the population come from Black and Minority Ethnic Groups. (55) It was the 11th most deprived local authority in England in the Indices of

For peer review only

Deprivation 2015. (56) The study will be conducted with adult migrants (>18 years) predominantly from the DRC and with local clinical, public health and community stakeholders based in Hackney. Specific inclusion and exclusion criteria are described in Table 1. There are an estimated 23,000 migrants from the DRC and Republic of Congo combined living in the UK, with Hackney hosting one of the largest communities. (57)

Table 1. Inclusion and exclusion criteria.

Target population	Inclusion criteria	Exclusion criteria
Migrants	• Born in the Democratic Republic of the Congo (Congo-Kinshasa), Republic of Congo (Congo-Brazzaville), Angola, or another Lingala-speaking region of Central Africa.<br>• Aged 18 or above.<br>• Currently residing in the UK.<br>• Willing and able to give informed consent.	• Not migrant as per earlier definition.<br>• Not born in one of the specified countries/regions.<br>• Below the age of 18.<br>• Temporarily in the UK for holiday, visiting friends/family or other reasons.<br>• Individuals who may lack the capacity to consent, as determined by the mental capacity act framework.
Local stakeholders	• Aged 18 or above.<br>• Volunteer or employee of a local group, organisation or business that has a vested interest in the health of the target community, such as local government, public health, National Health Service (NHS), community, and faith-based organisations.<br>• Willing and able to give informed consent.	• Not a local stakeholder as per earlier definition.<br>• Below the age of 18.<br>• Individuals who may lack the capacity to consent, as determined by the mental capacity act framework.

Study team and coalition

A coalition was formed in November 2021 to steer the study including 3 members of Hackney Congolese Women Support Group (HCWSG) (LML, LMK, SN - 2 Congolese migrant women and 1 British woman of Congolese descent), 1 network coordinator from Hackney Council for Voluntary Service (HCVS) (CH, a British woman with a Master’s in Community Engagement and extensive community and voluntary sector experience), 1 lead researcher from St George’s, University of London (AFC, a White migrant woman with an MSc in Control Infectious Diseases and extensive experience in social and behaviour change communication and implementation research), and 3 other academic co-researchers who provide an advisory function. The researchers will facilitate the involvement of the coalition members, providing research training and helping them to understand

For peer review only

and contribute to the research process. HCWSG will facilitate engagement with the local Congolese community and HCVS will facilitate relationships with the local integrated care system (a collaborative partnership between the organisations that deliver health and care needs locally) and voluntary and community sector.

Support for partners

Study partners from HCWSG and HCVS were financially compensated for their time and effort (58). All study resources and expenses were paid for by the project budget managed by the St George's research group. Non-financial contributions to HCWSG included honorary membership to the School of Oriental and African Studies (SOAS) Library, London (as requested to access African and Congolese literature to support their community work), training and upskilling opportunities, and grantwriting support.

Planning

In November-January 2021, the coalition had three 2-hour meetings to assign roles and responsibilities, plan the study, map the target population and stakeholders, and refine the research questions and approach, one half-day training session on qualitative interviewing techniques, led by AFC, and one half-day session to practice, pilot test and refine the interview topic guide. Based on the community's preference for oral communication and face-to-face interactions, the coalition decided that the study should be promoted mostly by word of mouth and flyers co-designed by the coalition, and that data from the Congolese community should be collected face-to-face (COVID-19 restrictions permitting).

Recruitment

The study seeks to recruit approximately 30 migrants living in and around Hackney, London, UK, to participate in the semi-structured qualitative interviews, 6-8 migrants to participate in the co-design workshops, and approximately 4-6 local (to Hackney) stakeholders to participate in the key informant interviews and consensus workshops, although actual sample sizes will be guided by data saturation. Participants will be recruited through publicity among the coalition's networks (e.g., by email bulletins, word of mouth, community meetings and advertisements) and by additional snowball sampling techniques. Participants will be compensated according to NIHR guidance (58) and reasonable expenses (travel, childcare, etc) will be paid.

Data collection

For peer review only

The study data and data collection methods are described in Table 2. Interviews with migrants will be conducted by 4 members of the coalition in Lingala, French or English, depending on the participant's preference (LML, LMK, SN are trilingual; AFC speaks English and will use an interpreter as required). Interviews with local stakeholders will be conducted in English by the lead researcher; consensus workshops will be co-facilitated by the coalition in English and co-design workshops will be co-facilitated by the coalition in Lingala, French and English. Qualitative interview data will be collected iteratively with a pilot-tested topic guide, which will be revised and refined as data are generated. Discussions with the interviewers will take place regularly to review the interview guide and data collection.

Table 2. Study activities, data, and data collection methods.

No.	Activity	Population	Data generated/collected	Data collection methods
1	Community days (n~3)	Congolese migrants (n~30) living in and around Hackney, London, UK	• Beliefs and experiences related to vaccination<br>• Suggestions for engagement approaches and interventions<br>• Community values<br>• Sociodemographic information	• IDIs (n~30)<br>• Post-it notes/interactive posters<br>• Sociodemographic surveys
2	Key informant interviews	Local clinical, public health and community stakeholders (n~6)	• Role and relationship with the Congolese community<br>• Description of local pathways, processes, and services<br>• Suggestions for potential interventions and considerations for implementation	• IDIs
3	Consensus workshops (n~2)	Local clinical, public health and community stakeholders (n~6)	• Insight and evidence-based discussion to generate feedback and suggestions for intervention design	• Consensus workshops
4	Co-design workshops (n~2)	Congolese migrants (n~8)	• Feedback and iteration on intervention prototypes	• Participatory workshops
n/a	Evaluation	All populations plus community coalition	• Feedback on involvement in co-design process<br>• Feedback on participation in study	• Evaluation forms/questionnaires<br>• Voting

For peer review only

			activities (IDIs, workshops)<br>• Feedback on final prototype	

Activity 1: Approximately 30 semi-structured, in-depth qualitative interviews with Congolese migrants will be conducted by the coalition to explore beliefs, perceptions and experiences relating to catch-up vaccination for routine vaccinations including MMR, Td/IPV, HPV, flu, and COVID-19 vaccination, and obtain suggestion for novel interventions. These will be delivered through "community days" held at HCVS, which is closely located to the local market that the Congolese community attend for their weekly shopping. Community days were planned to coincide with market days to facilitate attendance. To date, two community days have been held at HCVS, with interviews conducted in private rooms and a central social area provided for the community to gather over Congolese food and music. Additional data and insight about Congolese culture and values were collected through interactive posters in the social space. Post-interview evaluation forms and sociodemographic surveys were also collected. Information about other local community services (e.g. educational classes) were provided and referrals were facilitated by the HCVS coalition member.

Activity 2: Approximately 4-6 in-depth, online interviews will be conducted with local key informants/stakeholders (e.g. local GPs/nurses, clinical and public health staff, religious leaders and relevant community organisations in Hackney), to explore their role and relationship with the Congolese community, understand local pathways, processes and services and discuss potential interventions and considerations for implementation.

Activity 3: 1-2 online consensus workshops will be conducted with local stakeholders (from activity 2) to discuss emerging findings and obtain feedback and suggestions to inform ongoing data collection and design of interventions.

Activity 4: Approximately 2 co-design workshops will be conducted in-person with two groups of 4-6 Congolese migrants who participated in the in-depth interviews (activity 1) to discuss and iterate on the intervention functions that were selected by the coalition following data synthesis and appraisal and create an intervention prototype.

For peer review only

Evaluation: Activities will be evaluated with feedback from participants and community feedback on the final intervention prototype will be sought at the celebration event.

Analysis and preparation of initial intervention prototypes

Qualitative interview and consensus workshop data will be analysed iteratively, using Thematic Analysis (59), in NVivo software (Mac version). Anonymised digital recordings will be translated into English and transcribed verbatim by an independent professional translator, and transcripts, field notes, anonymous evaluation forms and other data collected during the activities (post-it notes, posters) will be imported into NVivo for coding and analysis. Sociodemographic data will be entered into Excel, aggregated, and summarised using descriptive statistics. Qualitative and quantitative analysis will be led by SGUL researchers, in consultation with the coalition to discuss, member-check, triangulate and interpret findings and define emergent themes. Themes will be mapped to the TDF (60) and BCW (61) to identify behavioural components and potential intervention functions (defined as broad categories of means by which an intervention can change behaviour) needed to change behaviour (42). Following data synthesis, interpretation and analysis from activities 1-3, the lead researcher will prepare a short summary of key findings and the corresponding intervention functions identified from the BCW to present to and verify with the coalition. The coalition will consider the candidate intervention functions using the APEASE criteria (affordability, practicability, effectiveness/cost effectiveness, acceptability, side-effects/safety, equity) (42), discuss potential interventions employing these functions that could be effective and tailored to the target population, and decide by consensus on approximately 2 intervention functions to take forward to the co-design workshops with Congolese community members. These intervention functions will be the starting point for the workshops, and potential intervention strategies involving these functions will be discussed, iterated on, and tailored with the participation of the community, with the end goal being to co-produce a single, detailed intervention prototype. Any summary notes from the workshops and photographs of visual data generated (e.g. post-it notes, illustrations, etc) will subsequently be imported into NVivo software for data management and further analysis by the coalition.

Schedule

The planned duration of the study is 12 months, starting from November 2021 and ending in November 2022.

Patient and public involvement

The initial idea for this study was informed by informal scoping workshops with diverse migrant community representatives and community-based organisations in London (predominantly City & Hackney) that were conducted by St George's, University of London in collaboration with Hackney CVS in January-March 2021 and published (2, 3). These participants will be invited to a roundtable discussion of findings and a dissemination event at the end of the study. The coalition was established to co-design and deliver the study. The study name was chosen by the coalition in consultation with the community to reflect the project's ethos of providing a platform for meaningful conversations around vaccination in the Congolese community. The planned consensus workshops with local stakeholders (activity 3) and co-design workshops with Congolese migrants (activity 4) will directly involve members of the public in designing tailored intervention prototypes. An independent patient and public involvement board (St George's Migrant Health Research Group NIHR Project Board) comprising 5 adult migrants with lived experience of accessing healthcare in the UK will also be consulted at significant points over the course of the study.

Ethics and dissemination: This study has been given favourable ethical opinion by the St George's University Research Ethics Committee (REC reference 2021.0128). A celebration event and webinar for participants, the local community and key stakeholders will be organised at the end of the study. The study findings will be widely disseminated at local, national and international levels, including conferences, policy and stakeholder meetings, voluntary and community sector assemblies, peer-reviewed journals, a PhD thesis, and multimedia outputs, e.g. video clips and tweets. Research data and outputs will be stored in the St George's Research Data Repository. Recommendations for a future larger scale study and testing of prototyped interventions will be made.

Acknowledgments: We thank the Hackney Congolese Women Support Group, Hackney Refugee and Migrant Forum and Hackney CVS, members of St George's Migrant Health Research Group NIHR Project Board, and the community representatives and community organisations consulted during our pre-engagement work. We thank all the participants in our study and in particular the Congolese communities in Northeast London.

Authors' contributions: AFC wrote the first draft, with input from SH and ASF, and all authors reviewed and commented on a final version.

Funding statement: This work was supported by the National Institute for Health Research (NIHR Advanced Fellowship 300072). SH and AFC are additionally funded by the Academy of Medical

For peer review only

Sciences (SBF005\1111) and the World Health Organization. SH acknowledges funding from the Novo Nordisk Foundation/La Caixa Foundation (Mobility– Global Medicine and Health Research grant). The funders did not have any direct role in the writing or decision to submit this manuscript for publication. The views expressed are those of the author(s) and not necessarily those of the NHS, the NIHR, or the Department of Health and Social Care. The funder of the study had no role in study design, data collection, data analysis, data interpretation, or writing of the report.

Competing interests statement: None declared.

Word Count: 3787/4000.

References

1. Crawshaw AF, Farah Y, Deal A, Rustage K, Hayward SE, Carter J, et al. Defining the determinants of under-vaccination in migrant populations in Europe to improve routine and COVID-19 vaccine uptake: a systematic review. . The Lancet Infectious Diseases (Accepted). 2021.
2. Crawshaw AF, Deal A, Rustage K, Forster AS, Campos-Matos I, Vandrevala T, et al. What must be done to tackle vaccine hesitancy and barriers to COVID-19 vaccination in migrants? Journal of Travel Medicine. 2021;28(4):taab048.
3. Crawshaw A, Farah Y, Deal A, Goldsmith L, Carter J, Rustage K, et al. Analysing drivers of routine and COVID-19 vaccination in migrants to develop tailored interventions. European Journal of Public Health. 2021;31(Supplement_3):ckab164. 248.
4. Public Health England. Vaccination of individuals with uncertain or incomplete immunisation status. Updated 26th August 2021. Available from: https://www.gov.uk/government/publications/vaccination-of-individuals-with-uncertain-or-incomplete-immunisation-status/vaccination-of-individuals-with-uncertain-or-incomplete-immunisation-status).
5. Jablonka A, Happle C, Grote U, Schleenvoigt BT, Hampel A, Dopfer C, et al. Measles, mumps, rubella, and varicella seroprevalence in refugees in Germany in 2015. Infection. 2016;44(6):781-7.
6. Mipatrini D, Stefanelli P, Severoni S, Rezza G. Vaccinations in migrants and refugees: a challenge for European health systems. A systematic review of current scientific evidence. Pathogens and Global Health. 2017;111(2):59-68.
7. Norman FF, Comeche B, Martínez-Lacalzada M, Pérez-Molina J-A, Gullón B, Monge-Maillo B, et al. Seroprevalence of vaccine-preventable and non-vaccine-preventable infections in migrants in Spain. Journal of Travel Medicine. 2021;28(4).
8. Charania NA, Gaze N, Kung JY, Brooks S. Vaccine-preventable diseases and immunisation coverage among migrants and non-migrants worldwide: A scoping review of published literature, 2006 to 2016. Vaccine. 2019;37(20):2661-9.
9. Deal A, Hayward SE, Huda M, Knights F, Crawshaw AF, Carter J, et al. Strategies and action points to ensure equitable uptake of COVID-19 vaccinations: A national qualitative interview study to explore the views of undocumented migrants, asylum seekers, and refugees. J Migr Health. 2021;4:100050.
10. Wilson L, Rubens-Augustson T, Murphy M, Jardine C, Crowcroft N, Hui C, et al. Barriers to immunization among newcomers: A systematic review. Vaccine. 2018;36(8):1055-62.
11. Vandrevala T, Hendy J, Hanson K, Alidu L, Ala A. Unpacking COVID-19 and Conspiracy Theories in the UK Black Community. medRxiv. 2022:2022.02.12.22270438.
12. Hargreaves S, Nellums LB, Ravensbergen SJ, Friedland JS, Stienstra Y, On Behalf Of The ESGITM Working Group On Vaccination In Migrants. Divergent approaches in the vaccination of recently arrived migrants to Europe: a survey of national experts from 32 countries, 2017. Eurosurveillance. 2018;23(41).
13. De Vito E, Parente P, de Waure C, Poscia A, Ricciardi W. A review of evidence on equitable delivery, access and utilization of immunization services for migrants and refugees in the WHO European Region. Copenhagen: World Health Organization. Regional Office for Europe; 2017 2017. Report No.: 9789289052740 2227-4316.
14. WHO. European Immunization Agenda 2030. Geneva: WHO, 2021. .

For peer review only

15. WHO. Leave no one behind. Guidance for planning and implementing catch-up vaccination. Geneva: WHO, 2021. .
16. European Centre for Disease Prevention and Control. Public health guidance on screening and vaccination for infectious diseases in newly arrived migrants within the EU/EEA. 2018.
17. Public Health England. Vaccination of individuals with uncertain or incomplete immunisation status 2021.
18. Carter J MA, Knights F, et al. . 'We don't routinely check vaccination background in adults': A national qualitative study of barriers and facilitators to vaccine delivery and uptake in adult migrants through UK primary care. . 2022 (pre-print).
19. Nguyen LH, Joshi AD, Drew DA, Merino J, Ma W, Lo C-H, et al. Self-reported COVID-19 vaccine hesitancy and uptake among participants from different racial and ethnic groups in the United States and United Kingdom. Nature Communications. 2022;13(1):636.
20. Kaiser Family Foundation. Latest Data on COVID-19 Vaccinations by Race/Ethnicity 2022 [Available from: https://www.kff.org/coronavirus-covid-19/issue-brief/latest-data-on-covid-19-vaccinations-by-race-ethnicity/.
21. Gaughan CH, Razieh C, Khunti K, Banerjee A, Chudasama YV, Davies MJ, et al. COVID-19 vaccination uptake amongst ethnic minority communities in England: a linked study exploring the drivers of differential vaccination rates. Journal of Public Health. 2022.
22. Crawshaw AF, et al. Defining the determinants of vaccine uptake and under-vaccination in migrant populations in Europe to improve routine and COVID-19 vaccine uptake: a systematic review. 2022 (Accepted, Lancet Infectious Diseases). https://www.medrxiv.org/content/10.1101/2021.11.08.21266058v1.
23. Quinn SC, Andrasik MP. Addressing Vaccine Hesitancy in BIPOC Communities — Toward Trustworthiness, Partnership, and Reciprocity. New England Journal of Medicine. 2021;385(2):97-100.
24. Williams SR, Driscoll AJ, LeBuhn HM, Chen WH, Neuzil KM, Ortiz JR. National routine adult immunisation programmes among World Health Organization Member States: an assessment of health systems to deploy COVID-19 vaccines. Eurosurveillance. 2021;26(17):2001195.
25. Momplaisir F, Haynes N, Nkwihoreze H, Nelson M, Werner RM, Jemmott J. Understanding Drivers of Coronavirus Disease 2019 Vaccine Hesitancy Among Blacks. Clin Infect Dis. 2021;73(10):1784-9.
26. Balasuriya L, Santilli A, Morone J, Ainooson J, Roy B, Njoku A, et al. COVID-19 Vaccine Acceptance and Access Among Black and Latinx Communities. JAMA Network Open. 2021;4(10):e2128575-e.
27. Goldsmith LP, Rowland-Pomp M, Hanson K, Deal A, Crawshaw AF, Hayward SE, et al. The use of social media platforms by migrant and ethnic minority populations during the COVID-19 pandemic: a systematic review. medRxiv. 2022:2022.02.07.22270579.
28. Armocida B, Formenti B, Missoni E, D'Apice C, Marchese V, Calvi M, et al. Challenges in the equitable access to COVID-19 vaccines for migrant populations in Europe. The Lancet Regional Health – Europe. 2021;6.
29. Bevan DotW. Vaccine Confidence Toolkit: Key findings from a scoping exercise exploring vaccine confidence in refugee, asylum seeker and undocumented migrant populations. . 2021.
30. Abba-Aji M, Stuckler D, Galea S, McKee M. Ethnic/racial minorities' and migrants' access to COVID-19 vaccines: A systematic review of barriers and facilitators. J Migr Health. 2022;5:100086.
31. National Institute for Health and Care Excellence (NICE). Shared learning database: Black Women in Health (BWIH): COVID-19 vaccine hesitancy – debunking the myths using a community engagement approach underpinned by NICE guidance. March 2021. 2021 [This example describes how GP's from Black Women in Health (BWIH) reduced COVID-19 vaccine hesitancy
amongst the BAME population by debunking the myths about COVID- vaccines by organising webinars, virtual group talks, podcasts, videos in other languages and dialects.]. Available from: https://www.nice.org.uk/sharedlearning/covid-19-vaccine-hesitancy-debunking-the-myths-using-a-community-engagement-approach-underpinned-by-nice-guidance.
32. (NIHR) NIfHR. Multimillion investment in new research projects to investigate higher COVID-19 risk among certain ethnic groups 2021 [Available from: https://www.nihr.ac.uk/news/multimillion-investment-in-new-research-projects-to-investigate-higher-covid-19-risk-among-certain-ethnic-groups/25333.
33. Greater London Authority. Translated COVID-19 Guidance 2021 [updated 2022. Available from: https://www.london.gov.uk/coronavirus/translated-covid-19-guidance.
34. Doctors of the World. Translated health information for patients 2022 [Available from: https://www.doctorsoftheworld.org.uk/translated-health-information/.
35. Doctors of the World. Vaccine confidence toolkit. 2021 [Available from: https://www.doctorsoftheworld.org.uk/what-we-stand-for/supporting-medics/vaccine-confidence-toolkit/.
36. Rustage K, Crawshaw A, Majeed-Hajaj S, Deal A, Nellums L, Ciftci Y, et al. Participatory approaches in the development of health interventions for migrants: a systematic review. BMJ Open. 2021;11(10):e053678.

For peer review only

37. World Health Organization Regional Office for Europe. Delivery of immunization services for refugees and migrants. Copenhagen: WHO Regional Office for Europe (Technical guidance on refugee and migrant health); 2019.
38. European Centre for Disease Prevention and Control. Public health guidance on screening and vaccination of migrants in the EU/EEA. Stockholm: ECDC; 2018.
39. Minkler M, Wallerstein N. Community-based participatory research for health: From process to outcomes: John Wiley & Sons; 2011.
40. Batalden M, Batalden P, Margolis P, Seid M, Armstrong G, Opipari-Arrigan L, et al. Coproduction of healthcare service. BMJ Quality & Safety. 2016;25(7):509-17.
41. Filipe A, Renedo A, Marston C. The co-production of what? Knowledge, values, and social relations in health care. PLoS Biol. 2017;15(5):e2001403-e.
42. Michie S AL, West R. . The Behaviour Change Wheel: A Guide to Designing Interventions. London: Silverback Publishing; 2014.
43. Garbutt JM, Dodd S, Walling E, Lee AA, Kulka K, Lobb R. Theory-based development of an implementation intervention to increase HPV vaccination in pediatric primary care practices. Implementation Science. 2018;13(1):45.
44. Roura M, Dias S, LeMaster JW, MacFarlane A. Participatory health research with migrants: Opportunities, challenges, and way forwards. Health Expect. 2021;24(2):188-97.
45. World Health Organization. Tailoring Immunization Programmes (TIP). Copenhagen: WHO EURO; 2019.
46. O'Mara-Eves A, Brunton G, Oliver S, Kavanagh J, Jamal F, Thomas J. The effectiveness of community engagement in public health interventions for disadvantaged groups: a meta-analysis. BMC Public Health. 2015;15(1):129.
47. Jarrett C, Wilson R, O'Leary M, Eckersberger E, Larson HJ. Strategies for addressing vaccine hesitancy - A systematic review. Vaccine. 2015;33(34):4180-90.
48. Vandrevala T, Alidu L, Hendy J, Shafi S, Ala A. 'It's possibly made us feel a little more alienated': How people from ethnic minority communities conceptualise COVID-19 and its influence on engagement with testing. Journal of Health Services Research & Policy.0(0):13558196211054961.
49. NHS Digital Screening and Immunisations Team. Childhood vaccination coverage statistics: local authorities all vaccinations. Hackney and City of London. 2020-2021. Updated September 2021. 2021 [Available from: https://app.powerbi.com/view?r=eyJrIjoiZTI3NWZhNzItMTIyZS00OWM2LTg0MzMtOGY5YTJjMGY0MjI1IiwidCI6IjUwZjYwNzFmLWJiZmUtNDAxYS04ODAzLTY3Mzc0OGU2MjllMiIsImMiOjh9.
50. Office for National Statistics - Public Health Data Asset NIMS. Age-standardised proportion of people aged 18 years and over who had received three vaccinations, by ethnic group, England: 16 September to 31 December 2021. 2022 [Available from: https://www.ons.gov.uk/peoplepopulationandcommunity/healthandsocialcare/conditionsanddiseases/articles/coronaviruscovid19latestinsights/vaccines.
51. Migration Policy Institute. Democratic Republic of the Congo: A Migration History Marked by Crises and Restrictions 2016 [Available from: https://www.migrationpolicy.org/article/democratic-republic-congo-migration-history-marked-crises-and-restrictions.
52. Deal A HS, Crawshaw AF, et al. . The immunisation status of UK-bound refugees, January 2018-October 2019: A restrospective population-based cross-sectional study. . 2022 (Unpublished).
53. Migration Observatory. Asylum and refugee resettlement in the UK. 11 May 2021. 2021 [Available from: https://migrationobservatory.ox.ac.uk/resources/briefings/migration-to-the-uk-asylum/.
54. Hasso Platner Institute of Design at Stanford University. An Introduction to Design Thinking: PROCESS GUIDE.
55. Office for National Statistics ; National Records of Scotland ; Northern Ireland Statistics and Research Agency (2017): 2011 Census aggregate data. UK Data Service (Edition: February 2017). DOI: http://dx.doi.org/10.5257/census/aggregate-2011-2
56. Hackney Council. Hackney Council Population Information: Deprivation data: Indices of Multiple Deprivation 2015 Briefing. 2015 [Available from: https://hackney.gov.uk/population.
57. (ONS) OfNS. Population of the UK by country of birth and nationality: individual country data: July 2020- June 2021. 25 November 2021. Online. 2021 [Available from: https://www.ons.gov.uk/peoplepopulationandcommunity/populationandmigration/internationalmigration/datasets/populationoftheunitedkingdombycountryofbirthandnationalityunderlyingdatasheets.
58. NIHR. Payment guidance for members of the public considering involvement in research. 2021 [Available from: https://www.nihr.ac.uk/documents/payment-guidance-for-members-of-the-public-considering-involvement-in-research/27372.

For peer review only

59. Braun V, Clarke V. Using thematic analysis in psychology. Qualitative Research in Psychology. 2006;3(2):77-101.
60. Cane J, O'Connor D, Michie S. Validation of the theoretical domains framework for use in behaviour change and implementation research. Implementation Science. 2012;7(1):37.
61. Michie S, van Stralen MM, West R. The behaviour change wheel: A new method for characterising and designing behaviour change interventions. Implementation Science. 2011;6(1):42.

For peer review only

Figure legend

Figure 1. Study process and activities, mapped to the 4 design thinking phases of empathise (with target population); define (target population's needs, their problems and your insights); ideate (by challenging assumptions and creating ideas for innovative solutions); prototype (to start creating solutions). (54)

For peer review only

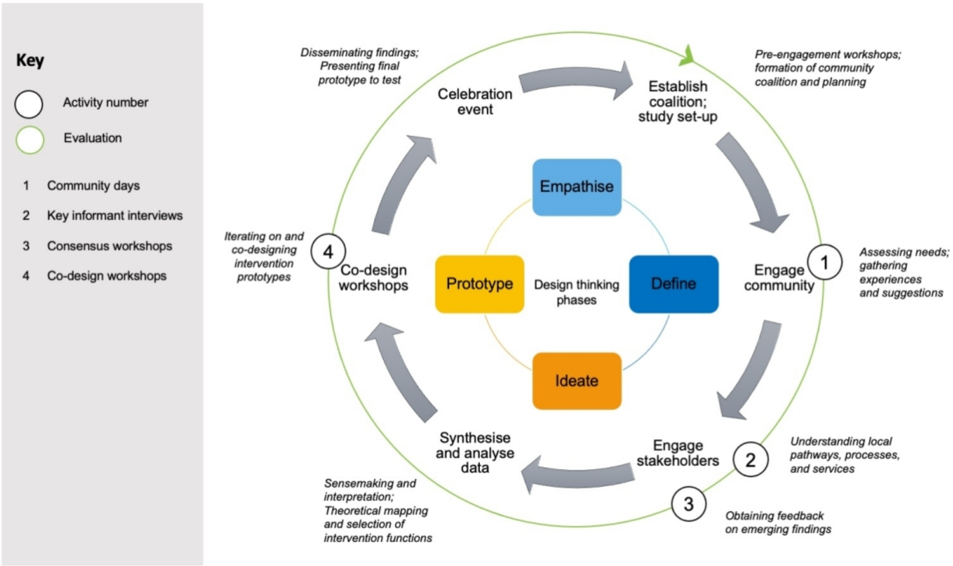

Figure 1. Study process and activities, mapped to the 4 design thinking phases of empathise (with target population); define (target population's needs, their problems and your insights); ideate (by challenging assumptions and creating ideas for innovative solutions); prototype (to start creating solutions). (54)

338x190mm (118 x 118 DPI)

Completed SRQR Reporting Checklist

No.	Topic	Item	Included in study protocol?
	**Title and abstract**		
S1	Title	Concise description of the nature and topic of the study Identifying the study as qualitative or indicating the approach (e.g., ethnography, grounded theory) or data collection methods (e.g., interview, focus group) is recommended	Yes
S2	Abstract	Summary of key elements of the study using the abstract format of the intended publication; typically includes background, purpose, methods, results, and conclusions	Yes
	**Introduction**		
S3	Problem formulation	Description and significance of the problem/phenomenon studied; review of relevant theory and empirical work; problem statement	Yes
S4	Purpose or research question	Purpose of the study and specific objectives or questions	Yes
	**Methods**		
S5	Qualitative approach and research paradigm	Qualitative approach (e.g., ethnography, grounded theory, case study, phenomenology, narrative research) and guiding theory if appropriate; identifying the research paradigm (e.g., postpositivist, constructivist/ interpretivist) is also recommended; rationale[b]	Yes
S6	Researcher characteristics and reflexivity	Researchers' characteristics that may influence the research, including personal attributes, qualifications/experience, relationship with participants, assumptions, and/or presuppositions; potential or actual interaction between researchers' characteristics and the research questions, approach, methods, results, and/or transferability	Yes
S7	Context	Setting/site and salient contextual factors; rationale[b]	Yes
S8	Sampling strategy	How and why research participants, documents, or events were selected; criteria for deciding when no further sampling was necessary (e.g., sampling saturation); rationale[b]	Yes
S9	Ethical issues pertaining to human subjects	Documentation of approval by an appropriate ethics review board and participant consent, or explanation for lack thereof; other confidentiality and data security issues	Yes
S10	Data collection methods	Types of data collected; details of data collection procedures including (as appropriate) start and stop dates of data collection and analysis, iterative process, triangulation of sources/methods, and modification of procedures in response to evolving study findings; rationale[b]	Yes
S11	Data collection instruments and technologies	Description of instruments (e.g., interview guides, questionnaires) and devices (e.g., audio recorders) used for data collection; if/how the instrument(s) changed over the course of the study	Yes
S12	Units of study	Number and relevant characteristics of participants, documents, or events included in the study; level of participation (could be reported in results)	Yes
S13	Data processing	Methods for processing data prior to and during analysis, including transcription, data entry, data management and security, verification of data integrity, data coding, and anonymization/deidentification of excerpts	Yes
S14	Data analysis	Process by which inferences, themes, etc., were identified and developed, including the researchers involved in data analysis; usually references a specific paradigm or approach; rationale [b]	Yes
S15	Techniques to enhance trustworthiness	Techniques to enhance trustworthiness and credibility of data analysis (e.g., member checking, audit trail, triangulation); rationale[b]	Yes
	**Results/findings**		
S16	Synthesis and interpretation	Main findings (e.g., interpretations, inferences, and themes); might include development of a theory or model, or integration with prior research or theory	N/A
S17	Links to empirical data	Evidence (e.g., quotes, field notes, text excerpts, photographs) to substantiate analytic findings	N/A
	**Discussion**		
S18	Integration with prior work, implications, transferability, and contribution(s) to the field	Short summary of main findings; explanation of how findings and conclusions connect to, support, elaborate on, or challenge conclusions of earlier scholarship; discussion of scope of application/ generalizability; identification of unique contribution(s) to scholarship in a discipline or field	N/A
S19	Limitations	Trustworthiness and limitations of findings	Yes
	**Other**		
S20	Conflicts of interest	Potential sources of influence or perceived influence on study conduct and conclusions; how these were managed	Yes
S21	Funding	Sources of funding and other support; role of funders in data collection, interpretation, and reporting	Yes

[a]The authors created the SRQR by searching the literature to identify guidelines, reporting standards, and critical appraisal criteria for qualitative research; reviewing the reference lists of retrieved sources; and contacting experts to gain feedback. The SRQR aims to improve the transparency of all aspects of qualitative research by providing clear standards for reporting qualitative research.

[b]The rationale should briefly discuss the justification for choosing that theory, approach, method, or technique rather than other options available, the assumptions and limitations implicit in those choices, and how those choices influence study conclusions and transferability. As appropriate, the rationale for several items might be discussed together.

ACADEMIC MEDICINE

Standards for Reporting Qualitative Research: A Synthesis of Recommendations

O'Brien, Bridget C.; Harris, Ilene B.; Beckman, Thomas J.; Reed, Darcy A.; Cook, David A.

Academic Medicine89(9):1245-1251, September 2014.

doi: 10.1097/ACM.0000000000000388

Standards for Reporting Qualitative Research (SRQR)a

Wolters Kluwer

Copyright © 2022 by the Association of American Medical Colleges

BMJ Open

Co-designing an intervention to strengthen COVID-19 vaccine uptake in Congolese migrants in the UK (LISOLO MALAMU): a participatory qualitative study protocol

Journal:	*BMJ Open*
Manuscript ID	bmjopen-2022-063462.R1
Article Type:	Protocol
Date Submitted by the Author:	10-Oct-2022
Complete List of Authors:	Crawshaw, Alison; St George's University of London, Institute for Infection and Immunity<br>Hickey, Caroline; Hackney Council for Voluntary Service, Hackney Refugee and Migrant Forum<br>Lutumba, Laura Muzinga; Hackney Congolese Women Support Group<br>Kitoko, Lusau Mimi; Hackney Congolese Women Support Group<br>Nkembi, Sarah; Hackney Congolese Women Support Group<br>Knights, Felicity; St George's Hospital Medical School<br>Ciftci, Yusuf; Doctors of the World UK<br>Goldsmith, Lucy; St George's University of London, Infection and Immunity Research Institute, and Population Health Research Institute<br>Vandrevala, Tushna; Kingston University, Centre for Applied Health and Social Care Research, Faculty of Health, Social Care and Education<br>Forster, Alice S; Our Future Health<br>Hargreaves, Sally; St George's University of London, Institute for Infection and Immunity
**Primary Subject Heading**:	Research methods
Secondary Subject Heading:	Infectious diseases, Patient-centred medicine, Public health, Qualitative research
Keywords:	PUBLIC HEALTH, QUALITATIVE RESEARCH, PRIMARY CARE, COVID-19

SCHOLARONE™
Manuscripts

Title: Co-designing an intervention to strengthen COVID-19 vaccine uptake in Congolese migrants in the UK (LISOLO MALAMU): a participatory qualitative study protocol

Authors

Alison F Crawshaw acrawsha@sgul.ac.uk

Caroline Hickey caroline@hcvs.org.uk

Laura Muzinga Lutumba hcwsg@outlook.com

Lusau Mimi Kitoko hcwsg@outlook.com

Sarah Luti Nkembi hcwsg@outlook.com

Felicity Knights fknights@sgul.ac.uk

Yusuf Ciftci yusufciftci4@gmail.com

Lucy P Goldsmith lgoldsmi@sgul.ac.uk

Tushna Vandrevala t.vandrevala@kingston.ac.uk

Alice S Forster alice.forman@ourfuturehealth.org.uk

Sally Hargreaves s.hargreaves@sgul.ac.uk

Corresponding author

Sally Hargreaves

s.hargreaves@sgul.ac.uk

The Migrant Health Research Group, Institute for Infection and Immunity, St George's, University of London, Cranmer Terrace, London, SW17 0RE, UK

Author affiliations

The Migrant Health Research Group, Institute for Infection and Immunity, St George's, University of London, Cranmer Terrace, London, SW17 0RE, UK (AF Crawshaw, F Knights, LP Goldsmith, S Hargreaves)

Centre for Applied Health and Social Care Research, Faculty of Health, Social Care and Education, Kingston University, University of London, UK (T Vandrevala)

Hackney Refugee and Migrant Forum and Hackney CVS, The Adiaha Antigha Centre, 24-30 Dalston Lane, London, E8 3AZ (C Hickey)

For peer review only - http://bmjopen.bmj.com/site/about/guidelines.xhtml

Hackney Congolese Women Support Group, c/o Hackney CVS, The Adiaha Antigha Centre, 24-30 Dalston Lane, London, E8 3AZ (LM Lutumba, LM Kitoko, SL Nkembi)

Doctors of the World UK, London, E14 5AA, UK (Y Ciftci)

Our Future Health, New Bailey, 4 Stanley Street, Manchester, M3 5JL, UK (AS Forster)

For peer review only

Abstract

Introduction

Migrants positively contribute to host societies yet experience barriers to health and vaccination services and systems and are considered to be an under-immunised group in many European countries. The COVID-19 pandemic has highlighted stark inequities in vaccine uptake, with migrants facing barriers to information, vaccine services and lower vaccine confidence. A key challenge, therefore, is developing tailored vaccination interventions, services and systems which account for and respond to the unique drivers of vaccine uptake in different migrant populations. Participatory research approaches, which meaningfully involve communities in co-constructing knowledge and solutions, have generated considerable interest in recent years for those tasked with designing and delivering public health interventions. How such approaches can be used to strengthen initiatives for COVID-19 and routine vaccination merits greater consideration.

Methods and analysis

LISOLO MALAMU ('Good Talk') is a community-based participatory research study which uses qualitative and co-production methodologies to involve adult Congolese migrants in developing a tailored intervention to increase COVID-19 vaccine uptake. Led by a community-academic coalition, the study will involve i) semi-structured in-depth interviews with adult Congolese migrants (born in Democratic Republic of Congo, >18 years), ii) interviews with professional stakeholders, and iii) co-design workshops with adult Congolese migrants. Qualitative data will be analysed collaboratively using reflexive Thematic Analysis, and behaviour change theory will be used in parallel to support the co-production of interventions and make recommendations across socio-ecological levels. The study will run from approximately November 2021-November 2022.

Ethics and dissemination

Ethics approval was granted by the St George's University Research Ethics Committee (REC reference 2021.0128). Study findings will be disseminated to a range of local, national and international audiences, and a community celebration event will be held to show impact and recognise contributions. Recommendations for implementation and evaluation of prototyped interventions will be made.

Strengths and limitations of this study:

Strengths

For peer review only

- This study uses community-based participatory research approaches, which promote principles of inclusivity and power sharing, and an academic-community partnership ('study coalition'), including 3 members of the target Congolese population, was formed to co-design and deliver the study, and co-wrote this protocol.
- The main research topic of COVID-19 vaccination was driven by the desires and needs of the study population and interventions will be co-produced which are informed by lived experience, insider knowledge and perspectives.
- Because the study coalition involves members of the target population who will act as peer researchers, recruitment and research activities will be designed and conducted in ways (times, locations, formats, etc) that are appropriate for the target population, and may therefore be more acceptable and foster increased levels of trust, which can increase validity and likelihood of success of community-led interventions.
- Building trust between the local and wider community, stakeholders and academic partners was a continuous process which began prior to study conception.

Limitations

- SGUL were lead recipients of the funding, which inherently skewed the power balance between partners, but efforts were made to overcome this, e.g. by giving the Congolese-led community-based organisation control over the spending and use of funds and providing them with skills-based training in budget management.

Keywords: community-based participatory research; transients and migrants; health disparities; vaccination uptake; community-academic research partnerships; co-production; intervention design

Introduction

Migrants (defined here as foreign-born individuals, see Box 1) contribute positively to their host societies (1) but many continue to be excluded from health and vaccination services and systems worldwide, are considered to be an under-immunised group, and suffer worse health outcomes than the general population (1-5). This has been brought to light acutely during the COVID-19 pandemic, where migrants have been disproportionately represented in COVID-19 deaths and all-cause mortality (2, 6), although even before this migrants (particularly those from low- and middle-income countries) were known to be at risk of under-immunisation for routine vaccinations (4, 5, 7, 8) and involved in outbreaks of serious vaccine-preventable diseases, including measles (9). In

For peer review only - http://bmjopen.bmj.com/site/about/guidelines.xhtml

addition to these risks, many migrant and refugee populations have now been shown to be more reluctant to vaccinate for COVID-19 and to have lower uptake compared to the general population, where this has been measured (2, 9-14).

A current challenge is developing tailored vaccination interventions, services and systems which adequately respond to the needs of migrant populations (15, 16). Many governments didn't include migrants well in their national plans at the start of the pandemic or adequately tailor health information to their linguistic needs and cultural preferences (e.g. only 6% (3/47) of Council of Europe member states translated information on testing or healthcare entitlements into a foreign language (17)) (6, 17, 18). In the UK, funding was mobilised to increase engagement with specific ethnic minority groups reporting lower levels of COVID-19 vaccine intent or uptake, including through outreach activities and the development of culturally relevant health information and messages (19, 20). However, few initiatives have specifically focused on understanding drivers of uptake in migrants (which is critical to increase uptake), actively involved migrant populations in the co-production of vaccine interventions, or considered how rapid emergency outreach might erode trust with already disenfranchised groups who – until the pandemic – had felt largely ignored (21-23).

Migrants are extremely heterogeneous and their reasons for under-vaccination are variable, multiple and complex. Depending on their migration status and the influence of social determinants of health, these may include barriers before, during, and after migration. Our recent systematic review (24) confirmed that access barriers including language, literacy, communication, practical, legal, and service barriers, are particularly important barriers to vaccination for migrants in transit and host countries, and that specific factors including country of origin, having more recently migrated, and being an asylum seeker or refugee, could be determinants of under-immunisation in migrants. Stigma, discrimination, xenophobia and racism are known to impact on access to health services in these populations (1). Adult and adolescent migrants are also thought to be at risk of remaining under-vaccinated for routine vaccinations after migration due to a lack of guidance (or implementation of guidance) on offering catch-up vaccinations and because, unlike children, they are not routinely incorporated into vaccination programmes upon arrival in most European countries arrival, including the UK (25). Literature on COVID-19 vaccination barriers in migrants is more limited, but recent studies have pointed in particular to access barriers including language and communication issues, as well as as lack of confidence stemming from mistrust of government and

For peer review only - http://bmjopen.bmj.com/site/about/guidelines.xhtml

health authorities (26) and the influence of pervasive factors including structural racism, marginalisation and discrimination (9, 12, 26-30).

The World Health Organization's new Immunization Agenda 2030 (IA2030) (31) emphasises the need for ensuring equitable access to vaccination for all populations, and promotes integrating vaccination throughout the lifecourse and catching-up adolescent and adult migrants with missed vaccines, doses and boosters, including COVID-19 vaccines, to close immunisation gaps. The Regional Risk Communication and Community Engagement (RCCE) Interagency Working Group has also set out four strategic objectives for reducing the negative impacts of COVID-19, including that responses and strategies are community-led, data-driven, collaborative, and reinforce capacity and local solutions (32). Participatory approaches, including community-based participatory research (CBPR), promote these principles and emphasise inclusivity and power sharing in conducting research. They are likely to be more effective than traditional research approaches when working with underserved and marginalised individuals and populations who may have reason not to wish to trust or engage with institutions, because they lift perceived barriers to involvement (33, 34). Rather than doing research "on" populations, participatory research actively involves those affected by the issue being studied as equal partners in the research process, so that research is done "with" populations and value is given to the subjectivity of lived experience in the creation of knowledge. In this way, research is embedded within, conducted in collaboration with, and tailored to, a specific community or population (35, 36). The relevance of participatory approaches to migrant health research and strengthening vaccination services has been noted (16, 37) and evidence shows that interventions driven by insights from the communities they are designed to serve are more cost-effective and lead to better results for health behaviour outcomes than traditional interventions (38, 39). However, much research into migrant health is still driven largely by the interests of academics, policymakers and clinicians rather than by the communities directly affected by the issue being studied (37). Implemention of policies promoting the inclusion of migrants in decision making across countries is also inconsistent (33). A global systematic review of studies that used participatory approaches with migrants found important shortfalls, with few studies actively including migrants in all research stages, and generally poor reporting of how participatory research approaches were used and principles upheld (40).

Rather than addressing migrants as a single, homogeneous group or retrofitting public health initiatives originally designed for the general population, there is a need to actively involve specific migrant sub-populations in co-constructing knowledge about their lived experiences to inform the

For peer review only - http://bmjopen.bmj.com/site/about/guidelines.xhtml

design of more sensitive health and vaccination initiatives which adequately respond to their needs, if we are to tackle existing health inequalities. For addressing COVID-19 vaccination inequities specifically, there is a need for more nuanced research into the drivers of COVID-19 vaccine uptake within and between migrant populations to advance understanding in this field and translate knowledge to practical action and interventions which account for migrants' unique cultural identity, beliefs and perspectives. While COVID-19 is the focus of this paper, similar gaps and opportunities exist with regards to routine vaccinations and in other disease areas, which require urgent focus.

The aim of this study is to use CBPR approaches to involve a specific sub-population of migrants (in this case, adult Congolese migrants) in Hackney in the co-design of a tailored intervention to increase COVID-19 vaccine uptake. It seeks to i) gather information about the local, socio-cultural, and historical context, ii) understand adult Congolese migrants' attitudes, beliefs and experiences relating to vaccination in general, COVID-19 vaccination, and other lived experiences of UK healthcare and vaccination policies, iii) understand local pathways, processes and services, and considerations for implementation of interventions with professional stakeholders; and iv) co-design a tailored intervention to strengthen COVID-19 vaccine uptake with Congolese migrants, which can be formally evaluated.

Box 1.

There is no internationally agreed definition of 'migrant', but for the purpose of this protocol we have defined a migrant as a foreign-born individual. Much of the language used to talk about migrants (and other minoritised populations) in public health is influenced by extant literature, databases/national registers used in population health, and international policies, many of which use inconsistent or inappropriate definitions and groupings of migrants, or fail to record migrants at all. This language, and the limitations of existing data, are problematic and incongruent with a community-centred approach which seeks to redistribute power, address injustices and decolonise medical practices. In our introduction we discuss recent literature regarding vaccination uptake in individuals grouped varyingly by race, ethnic group, or migrant status, depending on the citing source, in order to provide context to the research problem. We recognise the limitations of these groupings and suggest higher standards will be essential in addressing the needs of diverse populations.

Methods and analysis

For peer review only - http://bmjopen.bmj.com/site/about/guidelines.xhtml

Context

Around 16,000 migrants from the Democratic Republic of Congo (DRC) are thought to live in the UK (41), many of whom fled conflict and political instability and came to the UK to seek protection beginning in the late 1980s and 1990s (42) and continuing to the present day (43). Literature about Congolese diaspora in the UK are scarce. In December 2020, just before the UK government began rolling out the first COVID-19 vaccines, Congolese leaders of a small community-based organisation supporting Congolese migrants in London, UK (Hackney Congolese Women Support Group), voiced concerns during a community forum that misinformation about the COVID-19 pandemic and COVID-19 vaccines was spreading within their community, sparking widespread confusion and fear, with a large proportion of the community reluctant to get vaccinated. To the best of our knowledge, at this time there were no published data on Congolese migrants' vaccination attitudes, behaviours, and beliefs in the UK.

Forming a collaboration

Prior to study conception, exploratory workshops were held with representatives from various refugee and migrant populations in City & Hackney, London, UK. These were co-led by an academic researcher (AFC) and a community coordinator (CH) and facilitated by existing relationships and trust between CH and the local community organisations supported by the Hackney Refugee and Migrant Forum (within Hackney CVS). 3 online meetings were held in December 2020 – February 2021 and refugee and migrant 'experts by experience' were invited to share their views and concerns regarding local unmet needs and discuss potential solutions and courses of action, with a particular focus on COVID-19. Hackney Congolese Women Support Group (LML, LMK, SN) were one of the local charities to attend and their participation led to further discussions about a potential research collaboration, particularly because of their small size and limited funding success to date and clear unmet needs in their community regarding the national COVID-19 response that they highlighted. The three organisations (St George's, University of London, Hackney CVS and Hackney Congolese Women Support Group) discussed potential ways of working together to identify solutions starting with the needs and desires of the target population, before deciding to form a partnership or 'coalition' to co-design and lead the study. All partners discussed relative experiences, expectations, goals, timelines and budget, and used their respective assets to increase understanding of the other coalition members. For example, AFC and CH helped to create understanding of possible research approaches and methods, ethics, rights and ownership and

For peer review only - http://bmjopen.bmj.com/site/about/guidelines.xhtml

empower the Congolese partners to participate with full voice; LML, LMK and SN advised on local preferences, customs and values that should be respected and provided valuable context on the lived experience of the target population.

Study coalition and reflexivity

The coalition includes 3 women with lived experience as a Congolese migrant or descendant in the target population in London (LML, LMK, SN) , 1 woman (CH) representing the local community and voluntary sector and 1 woman (AFC) representing academia. Each of the coalition members hold positions of both privilege and marginality and the influence of these positions with respect to each other and the target population will be considered reflexively throughout the course of the study. Although there are some shared characteristics between all members of the coalition, AFC and CH generally consider themselves 'outsiders' and LML, LMK and SN consider themselves 'insiders' to the study population.

Study planning

The coalition held three 2-hour planning meetings in November-January 2021 to agree roles, responsibilities, study aims and objectives and plan the study (e.g. recruitment, data collection, analysis, dissemination plans), with additional meetings to prepare and refine study tools. Further meetings and reflection sessions are planned. AFC led one half-day training session for the coalition on qualitative interviewing techniques and one half-day session to practice, pilot test and refine the interview topic guide. CH provided additional training on facilitation skills. The coalition chose a community-based participatory research (CBPR) approach, where partners are equals and actively involved in all stages of the research, and agreed to plan through an equity lens and prioritise building relationships and trust. The study was named 'Lisolo Malamu', meaning 'Good Talk' in Lingala (suggested by Hackney Congolese Women Support Group) to reflect the aim of facilitating dialogues and meaningful conversation around COVID-19 vaccination and other health topics with their community.

Study design

'Lisolo Malamu' ('Good Talk') is a CBPR study which uses co-production and qualitative research methods to engage Congolese migrants in developing a tailored intervention to increase vaccine

For peer review only - http://bmjopen.bmj.com/site/about/guidelines.xhtml

uptake. It involves 3 main activities: 1) community days, involving qualitative in-depth interviews and interactive posters with Congolese migrants, 2) in-depth interviews (IDIs) with local clinical, public health and community stakeholders, and 3) co-design workshops with Congolese migrants. The principles of design thinking (an iterative, solutions-based approach to problem-solving that starts with the needs and desires of the target population) (44) and behaviour change theory (45) will be used to support intervention co-development. An evaluation component will be embedded across all activities. The study process is illustrated in Figure 1. Good practices, challenges and facilitators relating to the implementation of the study and the method of using co-design will also be documented.

The study was co-designed by an academic-community partnership ('coalition') which includes 3 members of the Congolese target population (described earlier). The coalition co-wrote this protocol and will participate in all stages of the research and dissemination, including as peer researchers. This protocol reports on the decisions made regarding the study design to date and the full process will be written up at the end of the study.

[FIGURE 1]

Setting and population

The study is being carried out in Hackney, London, UK, a highly diverse London borough, in which more than 89 languages are spoken and around 40% of the population come from Black and Minority Ethnic Groups. (46) It was the 11th most deprived local authority in England in the Indices of Deprivation 2015. (47) The study will be conducted with adult migrants (>18 years) from the DRC and with local clinical, public health and community stakeholders based in Hackney. Specific inclusion and exclusion criteria are described in Table 1. Hackney is thought to host one of the largest communities of Congolese migrants in the UK. (48)

Table 1. Inclusion and exclusion criteria.

Target population	Inclusion criteria	Exclusion criteria
Migrants	• Born in the Democratic Republic of Congo (DRC).<br>• Aged 18 or above.<br>• Currently residing in the UK.	• Not migrant as per earlier definition.<br>• Not born in DRC.<br>• Below the age of 18.

For peer review only - http://bmjopen.bmj.com/site/about/guidelines.xhtml

For peer review only

	• Willing and able to give informed consent.	• Temporarily in the UK for holiday, visiting friends/family or other reasons.<br>• Individuals who may lack the capacity to consent, as determined by the mental capacity act framework.
Local stakeholders	• Aged 18 or above.<br>• Volunteer or employee of a local group, organisation or business that has a vested interest in the health of the target community, such as local government, public health, National Health Service (NHS), community, and faith-based organisations.<br>• Willing and able to give informed consent.	• Not a local stakeholder as per earlier definition.<br>• Below the age of 18.<br>• Individuals who may lack the capacity to consent, as determined by the mental capacity act framework.

Recruitment

The study seeks to recruit approximately 30 Congolese migrants living in and around Hackney, London, UK to participate in semi-structured qualitative interviews, 6-8 migrants to participate in the co-design workshops, and approximately 4-6 local (to Hackney) professional stakeholders to participate in the key informant interviews. Hackney Congolese Women Support Group will lead the recruitment of local migrants, using word-of mouth, flyers co-developed by the coalition, and additional snowball sampling techniques. Professional stakeholders will be recruited through publicity among the coalition's networks (e.g. email bulletins, word of mouth, local meetings, advertisements). Participants will be compensated according to NIHR guidance (49) and reasonable expenses (travel, childcare, etc) will be paid.

Data collection and activities

The study data and data collection methods are described in Table 2. Due to cultural preferences, data will be collected face-to-face (COVID-19 restrictions permitting). Translated participant information sheets will be distributed at least a week in advance of interviews, with participants given the chance to ask questions and decide whether they would like to participate. Written informed consent will be obtained in writing prior to starting the interview. Interviews with migrants will be conducted by 4 members of the coalition in Lingala, French or English, depending on the participant's preference (LML, LMK, SN are trilingual; AFC speaks English and will use an interpreter as required). Interviews with professional stakeholders will be conducted in English by AFC; co-design workshops will be co-facilitated by the coalition in Lingala, French and English. Qualitative

For peer review only - http://bmjopen.bmj.com/site/about/guidelines.xhtml

interview data will be collected with a semi-structured pilot-tested topic guide, which will be used flexibly. Interviewers will meet regularly during the data collection period to debrief on the interview process, discuss data, and adapt the topic guide if required.

Table 2. Study activities, data, and data collection methods.

No.	Activity	Population	Data generated/collected	Data collection methods	Person(s) responsible
1	Community days (n~3)	Congolese migrants (n~30) living in and around Hackney, London, UK	• Information about the local, socio-cultural and historical context, customs and preferences<br>• Beliefs and experiences related to routine and COVID-19 vaccination and other lived experiences of UK healthcare and vaccination policies<br>• Suggestions for engagement approaches and interventions<br>• Sociodemographic information	• IDIs (n~30)<br>• Post-it notes/interactive posters/graffiti walls<br>• Sociodemographic surveys	LML, LMK, SN and AFC will obtain informed consent and conduct IDIs. CH will manage logistics, registration, ensure participants are welcomed and comfortable, and support linkage to wraparound services.
2	Key informant interviews	Local clinical, public health and community stakeholders (n~6)	• Role and relationship with the Congolese community<br>• Description of local pathways, processes, and services<br>• Suggestions for potential interventions and considerations for implementation	• IDIs	AFC will obtain informed consent and conduct IDIs.
3	Co-design workshops (n~2)	Congolese migrants (n~8)	• Co-development of and iteration on intervention prototypes	• Participatory workshops	LML, LMK, SN, AFC and CH will facilitate workshops.
n/a	Evaluation	All populations plus community coalition	• Feedback on involvement in co-design process<br>• Feedback on participation in	• Evaluation forms/questionnaires<br>• Voting	CH will manage evaluation data with support from coalition.

For peer review only - http://bmjopen.bmj.com/site/about/guidelines.xhtml

			study activities (IDIs, workshops)<br>• Feedback on final prototype		

Activity 1: "Community days" will be held, during which approximately 30 semi-structured, in-depth qualitative interviews with Congolese migrants will be conducted to explore beliefs, perceptions and experiences relating to routine and COVID-19 vaccination, UK healthcare and policies, and obtain suggestions for novel vaccination interventions. Additional data and insights about the local, socio-cultural, and historical context, and Congolese culture, customs and preferences, will be collected through interactive posters in the social space. Post-interview evaluation forms and sociodemographic surveys will be collected. Information about local services (e.g. educational classes, housing) will be available and referrals will be facilitated by CH. Community days will be held at a community centre near a local market attended by many Congolese for their weekly shopping, and planned to coincide with market days to encourage attendance. At the time of submitting this protocol, two community days have been held, with interviews conducted in private rooms and a central social area provided for the community to gather over Congolese food and music.

Activity 2: Approximately 4-6 in-depth, online interviews will be conducted with local key informants/professional stakeholders (e.g. local GPs/nurses, clinical and public health staff, religious leaders and relevant community organisations in Hackney), to explore their role and relationship with the Congolese community, understand local pathways, processes and services and discuss potential interventions and considerations for implementation.

Activity 3: Approximately 2 co-design workshops will be conducted in person with two groups of 4-6 Congolese migrants who participated in the in-depth interviews (activity 1) to discuss and iterate on intervention functions and create an intervention prototype.

Evaluation: Activities will be evaluated with feedback from participants and community feedback on the final intervention prototype will be sought at the celebration event.

Data analysis and preparation of initial intervention prototypes

For peer review only - http://bmjopen.bmj.com/site/about/guidelines.xhtml

Qualitative interview and consensus workshop data will be analysed collaboratively by the coalition, to enhance understanding, interpretation and reflexivity (50), manually and in NVivo software (Mac version). Anonymised digital recordings will be translated into English and transcribed verbatim by an independent professional translator, and transcripts, field notes, anonymous evaluation forms and other data collected during the activities (post-it notes, posters) will be imported into Nvivo for coding and analysis. Sociodemographic data will be entered into Excel, aggregated, and summarised using descriptive statistics.

Analysis will take place in two stages, exploring both inductive and deductive orientations to data. The first stage will follow the six steps of reflexive Thematic Analysis (51, 52): 1) dataset familiarisation, 2) coding, 3) initial theme generation (whereby themes are patterns anchored by a shared idea, meaning or concept), 4) theme development and review, 5) theme refining, defining and naming, and 6) writing up (52). This stage will be more inductive. All members of the coalition will have access to the study data and will hold specific responsibilities to support the collaborative process: the academic researcher will manage the data and serve as a 'facilitator', guiding the coalition through the analytical steps to encourage and support their active participation. The community partners will facilitate member-checking the study data with participants and triangulation of sources. Reflexive Thematic Analysis was chosen because it values subjectivity in knowledge creation, helps locate personal experiences within wider socio-cultural contexts, and is suited to research that needs to generate practical and actionable outcomes (53).

The second stage will involve mapping the data to the Theoretical Domains Framework (TDF) (54) and Behaviour Change Wheel (BCW) (55) to identify behavioural components and potential intervention functions (defined as broad categories of means by which an intervention can change behaviour) needed to change behaviour (45). The comprehensive and practical TDF and BCW (45) were selected to guide intervention development because they were specifically developed for implementation research, and support identifying changes at individual, organisational and system level, and making policy recommendations. We expect this stage to be more deductive, with the analysis shaped by existing theoretical constructs. The compatibility of the two approaches will be critically discussed in the write up. Candidate intervention functions will be selected by the coalition using the APEASE criteria (affordability, practicability, effectiveness/cost effectiveness, acceptability, side-effects/safety, equity) (45), with approximately 2 suitable intervention functions taken forward to the co-design workshops with the Congolese study population. These intervention functions will be the starting point for the workshops, and potential intervention strategies involving these

For peer review only - http://bmjopen.bmj.com/site/about/guidelines.xhtml

functions will be discussed, iterated on, and tailored with the participation of the community, with the end goal being to co-produce a single, detailed intervention prototype. Any summary notes from the workshops and photographs of visual data generated (e.g. post-it notes, illustrations, etc) will subsequently be imported into Nvivo software for data management and further analysis by the coalition.

Schedule

The planned duration of the study is 12 months, starting from November 2021 and ending in November 2022.

Support for partners

Study partners from Hackney Congolese Women Support Group and Hackney CVS will be financially compensated for their time and effort (49). All study resources and expenses will be paid for by the project budget managed by the St George's research group. Non-financial contributions to Hackney Congolese Women Support Group include honorary library membership, training and upskilling opportunities, and grantwriting support.

Patient and public involvement

Patient and public involvement is embedded throughout the participatory study design and approach. An independent patient and public involvement board (St George's Migrant Health Research Group NIHR Project Board) comprising 5 adult migrants with lived experience of accessing healthcare in the UK will also be consulted at significant points over the course of the study.

Ethics and dissemination: This study was granted ethics approval by the St George's University Research Ethics Committee (REC reference 2021.0128). A celebration event and webinar for participants, the local community and professional stakeholders will be organised at the end of the study to show impact and recognise contributions. The study findings will be disseminated at local, national and international levels, including through conferences, policy, stakeholder and voluntary/community sector meetings, peer-reviewed journals, a PhD thesis, and multimedia outputs, e.g. video clips and tweets. Research data and outputs will be stored in the St George's Research Data Repository. Recommendations for a future larger scale study and testing of prototyped interventions will be made.

For peer review only - http://bmjopen.bmj.com/site/about/guidelines.xhtml

For peer review only

Acknowledgments: We thank the Hackney Congolese Women Support Group, Hackney Refugee and Migrant Forum and Hackney CVS, members of St George's Migrant Health Research Group NIHR Project Board, and the community representatives and community organisations consulted during our pre-engagement work. We thank all the participants in our study and in particular the Congolese migrant communities in Northeast London.

Authors' contributions:

AFC, SH, ASF, LML, SN, LMK and CH collectively discussed, conceived and co-designed the study and prepared all of the study documents (PIS, ICF, recruitment flyers, topic guides, surveys, etc). LML, SN and LMK recruited participants. AFC, LML, SN and LMK collected the data, and AFC, LML, SN, LMK and CH collaboratively analysed and interpreted the data, with input and comments from SH, ASF, YC, FK, TV and LG. AFC, LML, SN, LMK and CH organised and hosted the study celebration. AFC wrote the first draft, with input from the coalition (SH, ASF, LML, SN, LMK, CH), and all authors (AFC, SH, ASF, LML, SN, LMK, CH, YC, FK, TV, LG) reviewed and commented on a final version.

Funding statement: This work was supported by the National Institute for Health Research (NIHR Advanced Fellowship 300072). SH and AFC are additionally funded by the Academy of Medical Sciences (SBF005\1111) and the World Health Organization. SH acknowledges funding from the Novo Nordisk Foundation/La Caixa Foundation (Mobility– Global Medicine and Health Research grant). The funders did not have any direct role in the writing or decision to submit this manuscript for publication. The views expressed are those of the author(s) and not necessarily those of the NHS, the NIHR, or the Department of Health and Social Care. The funder of the study had no role in study design, data collection, data analysis, data interpretation, or writing of the report.

Competing interests statement: None declared.

Word Count: 3683/4000.

References

1. Abubakar I, Aldridge RW, Devakumar D, Orcutt M, Burns R, Barreto ML, et al. The UCL–Lancet Commission on Migration and Health: the health of a world on the move. The Lancet. 2018;392(10164):2606-54.

2. European Centre for Disease Prevention and Control. Reducing COVID-19 transmission and strengthening vaccine uptake among migrant populations in the EU/EEA – 3 June 2021. ECDC: Stockholm; 2021.

For peer review only - http://bmjopen.bmj.com/site/about/guidelines.xhtml

3. Hargreaves S Hayward S, Noori T, McKee M, Kumar B. COVID-19: counting migrants in. Lancet.
2021;398(10296):211–12. doi: 10.1016/S0140-6736(21)01339-8.

4. Mipatrini D, Stefanelli P, Severoni S, Rezza G. Vaccinations in migrants and refugees: a challenge for European health systems. A systematic review of current scientific evidence. Pathogens and Global Health. 2017;111(2):59-68.

5. Charania NA, Gaze N, Kung JY, Brooks S. Vaccine-preventable diseases and immunisation coverage among migrants and non-migrants worldwide: A scoping review of published literature, 2006 to 2016. Vaccine. 2019;37(20):2661-9.

6. Hayward SE, Deal A, Cheng C, Crawshaw A, Orcutt M, Vandrevala TF, et al. Clinical outcomes and risk factors for COVID-19 among migrant populations in high-income countries: A systematic review. J Migr Health. 2021;3:100041.

7. Jablonka A, Happle C, Grote U, Schleenvoigt BT, Hampel A, Dopfer C, et al. Measles, mumps, rubella, and varicella seroprevalence in refugees in Germany in 2015. Infection. 2016;44(6):781-7.

8. Norman FF, Comeche B, Martínez-Lacalzada M, Pérez-Molina J-A, Gullón B, Monge-Maillo B, et al. Seroprevalence of vaccine-preventable and non-vaccine-preventable infections in migrants in Spain. Journal of Travel Medicine. 2021;28(4).

9. Deal A, Hayward SE, Huda M, Knights F, Crawshaw AF, Carter J, et al. Strategies and action points to ensure equitable uptake of COVID-19 vaccinations: A national qualitative interview study to explore the views of undocumented migrants, asylum seekers, and refugees. J Migr Health. 2021;4:100050.

10. El Salibi N, Abdulrahim S, El Haddad M, Bassil S, El Khoury Z, Ghattas H et al. COVID-19 vaccine
acceptance in older Syrian refugees: preliminary findings from an ongoing study. Prev Med Rep. 2021;24:101606. doi: 10.1016/j.pmedr.2021.101606.

11. Community, engagement and accountability: survey on the beliefs of the vulnerable population
regarding the COVID-19 vaccine; a case study of the Hellenic Red Cross. Athens: Hellenic Red Cross; 2021 (https://communityengagementhub.org/resource/survey-on-the-beliefs-of-thevulnerable-population-regarding-the-covid-19-vaccine/, accessed 11 January 2022).

12. Armocida B, Formenti B, Missoni E, D'Apice C, Marchese V, Calvi M, et al. Challenges in the equitable access to COVID-19 vaccines for migrant populations in Europe. The Lancet Regional Health – Europe. 2021;6.

13. Führer A, Pacolli L, Yilmaz-Aslan Y, Brzoska P. COVID-19 Vaccine Acceptance and Its Determinants among Migrants in Germany—Results of a Cross-Sectional Study. Vaccines. 2022;10(8):1350.

14. McFadden SM, Demeke J, Dada D, Wilton L, Wang M, Vlahov D, et al. Confidence and Hesitancy During the Early Roll-out of COVID-19 Vaccines Among Black, Hispanic, and Undocumented Immigrant Communities: a Review. Journal of Urban Health. 2022;99(1):3-14.

For peer review only - http://bmjopen.bmj.com/site/about/guidelines.xhtml

15. World Health Organization. Strengthening COVID-19 vaccine demand and uptake in refugees and migrants: an operational guide to support all those responsible for planning and implementing the rollout of COVID-19 vaccine to refugees and migrants at national and local levels, 14 March 2022. Geneva: World Health Organization; 2022 2022. Contract No.: WHO/2019-nCoV/immunization/demand_planning/refugees_and_migrants/2022.1.

16. Declich S, De Ponte G, Marchetti G, Dente MG, Tosti ME, Tavoschi L, et al. Life-course vaccinations for migrants and refugees: Drawing lessons from the COVID-19 vaccination campaigns. J Glob Health. 2022;12:03064.

17. Nezafat Maldonado BM, Collins J, Blundell HJ, Singh L. Engaging the vulnerable: A rapid review of public health communication aimed at migrants during the COVID-19 pandemic in Europe. Journal of Migration and Health. 2020;1-2:100004.

18. Kondilis E, Papamichail D, McCann S, Carruthers E, Veizis A, Orcutt M, et al. The impact of the COVID-19 pandemic on refugees and asylum seekers in Greece: A retrospective analysis of national surveillance data from 2020. EClinicalMedicine. 2021;37:100958.

19. National Institute for Health and Care Excellence (NICE). Shared learning database: Black Women in Health (BWIH): COVID-19 vaccine hesitancy – debunking the myths using a community engagement approach underpinned by NICE guidance. March 2021. 2021 [This example describes how GP's from Black Women in Health (BWIH) reduced COVID-19 vaccine hesitancy amongst the BAME population by debunking the myths about COVID- vaccines by organising webinars, virtual group talks, podcasts, videos in other languages and dialects.]. Available from: https://www.nice.org.uk/sharedlearning/covid-19-vaccine-hesitancy-debunking-the-myths-using-a-community-engagement-approach-underpinned-by-nice-guidance.

20. (NIHR) NIfHR. Multimillion investment in new research projects to investigate higher COVID-19 risk among certain ethnic groups 2021 [Available from: https://www.nihr.ac.uk/news/multimillion-investment-in-new-research-projects-to-investigate-higher-covid-19-risk-among-certain-ethnic-groups/25333.

21. Etti M, Fofie H, Razai M, Crawshaw AF, Hargreaves S, Goldsmith LP. Ethnic minority and migrant underrepresentation in Covid-19 research: Causes and solutions. EClinicalMedicine. 2021;36.

22. Burgess RA, Osborne RH, Yongabi KA, Greenhalgh T, Gurdasani D, Kang G, et al. The COVID-19 vaccines rush: participatory community engagement matters more than ever. Lancet. 2021;397(10268):8-10.

23. Crawshaw AF, Deal A, Rustage K, Forster AS, Campos-Matos I, Vandrevala T, et al. What must be done to tackle vaccine hesitancy and barriers to COVID-19 vaccination in migrants? Journal of Travel Medicine. 2021;28(4):01.

24. Crawshaw AF, Farah Y, Deal A, Rustage K, Hayward SE, Carter J, et al. Defining the determinants of under-vaccination in migrant populations in Europe to improve routine and COVID-19 vaccine uptake: a systematic review. . The Lancet Infectious Diseases (Accepted). 2021.

25. Hargreaves S, Nellums LB, Ravensbergen SJ, Friedland JS, Stienstra Y, On Behalf Of The ESGITM Working Group On Vaccination In Migrants. Divergent approaches in the vaccination of

For peer review only

recently arrived migrants to Europe: a survey of national experts from 32 countries, 2017. Eurosurveillance. 2018;23(41).

26. Abba-Aji M, Stuckler D, Galea S, McKee M. Ethnic/racial minorities' and migrants' access to COVID-19 vaccines: A systematic review of barriers and facilitators. J Migr Health. 2022;5:100086.

27. Momplaisir F, Haynes N, Nkwihoreze H, Nelson M, Werner RM, Jemmott J. Understanding Drivers of Coronavirus Disease 2019 Vaccine Hesitancy Among Blacks. Clin Infect Dis. 2021;73(10):1784-9.

28. Balasuriya L, Santilli A, Morone J, Ainooson J, Roy B, Njoku A, et al. COVID-19 Vaccine Acceptance and Access Among Black and Latinx Communities. JAMA Network Open. 2021;4(10):e2128575-e.

29. Goldsmith LP, Rowland-Pomp M, Hanson K, Deal A, Crawshaw AF, Hayward SE, et al. The use of social media platforms by migrant and ethnic minority populations during the COVID-19 pandemic: a systematic review. medRxiv. 2022:2022.02.07.22270579.

30. Bevan DotW. Vaccine Confidence Toolkit: Key findings from a scoping exercise exploring vaccine confidence in refugee, asylum seeker and undocumented migrant populations. . 2021.

31. WHO. European Immunization Agenda 2030. Geneva: WHO, 2021. .

32. COVID-19 global risk communication and community engagement strategy: December 2020 –

May 2021. Geneva: Collective Service for Risk Communication and Community Engagement; 2020 (interim guidance 23 December 2020; https://rccehub.wpengine.com/wp-content/ uploads/2021/02/RCCE_GlobalStrategy-25012021.pdf, accessed 7 October 2022).

33. MacFarlane A, Ogoro M, de Freitas C, Niranjan V, Severoni S, Waagensen E. Migrants' involvement in health policy, service development and research in the WHO European Region: A narrative review of policy and practice. Tropical Medicine & International Health. 2021;26(10):1164-76.

34. Wallerstein NB, Duran B. Using Community-Based Participatory Research to Address Health Disparities. Health Promotion Practice. 2006;7(3):312-23.

35. Batalden M, Batalden P, Margolis P, Seid M, Armstrong G, Opipari-Arrigan L, et al. Coproduction of healthcare service. BMJ Quality & Safety. 2016;25(7):509-17.

36. Filipe A, Renedo A, Marston C. The co-production of what? Knowledge, values, and social relations in health care. PLoS Biol. 2017;15(5):e2001403-e.

37. Roura M, Dias S, LeMaster JW, MacFarlane A. Participatory health research with migrants: Opportunities, challenges, and way forwards. Health Expect. 2021;24(2):188-97.

38. World Health Organization. Tailoring Immunization Programmes (TIP). Copenhagen: WHO EURO; 2019.

39. O'Mara-Eves A, Brunton G, Oliver S, Kavanagh J, Jamal F, Thomas J. The effectiveness of community engagement in public health interventions for disadvantaged groups: a meta-analysis. BMC Public Health. 2015;15(1):129.

For peer review only - http://bmjopen.bmj.com/site/about/guidelines.xhtml

40. Rustage K, Crawshaw A, Majeed-Hajaj S, Deal A, Nellums L, Ciftci Y, et al. Participatory approaches in the development of health interventions for migrants: a systematic review. BMJ Open. 2021;11(10):e053678.

41. Office for National Statistics. Population of the UK by country of birth and nationality: individual country data. July 2020 to June 2021 edition. . 2021.

42. Migration Policy Institute. Democratic Republic of the Congo: A Migration History Marked by Crises and Restrictions 2016 [Available from: https://www.migrationpolicy.org/article/democratic-republic-congo-migration-history-marked-crises-and-restrictions.

43. Deal A, Hayward SE, Crawshaw AF, Goldsmith LP, Hui C, Dalal W, et al. Immunisation status of UK-bound refugees between January, 2018, and October, 2019: a retrospective, population-based cross-sectional study. The Lancet Public Health. 2022;7(7):e606-e15.

44. Hasso Platner Institute of Design at Stanford University. An Introduction to Design Thinking: PROCESS GUIDE.

45. Michie S AL, West R. . The Behaviour Change Wheel: A Guide to Designing Interventions. London: Silverback Publishing; 2014.

46. Office for National Statistics ; National Records of Scotland ; Northern Ireland Statistics and Research Agency (2017): 2011 Census aggregate data. UK Data Service (Edition: February 2017). DOI: http://dx.doi.org/10.5257/census/aggregate-2011-2

47. Hackney Council. Hackney Council Population Information: Deprivation data: Indices of Multiple Deprivation 2015 Briefing. 2015 [Available from: https://hackney.gov.uk/population.

48. (ONS) OfNS. Population of the UK by country of birth and nationality: individual country data: July 2020- June 2021. 25 November 2021. Online. 2021 [Available from: https://www.ons.gov.uk/peoplepopulationandcommunity/populationandmigration/internationalmigration/datasets/populationoftheunitedkingdombycountryofbirthandnationalityunderlyingdatasheets.

49. NIHR. Payment guidance for members of the public considering involvement in research. 2021 [Available from: https://www.nihr.ac.uk/documents/payment-guidance-for-members-of-the-public-considering-involvement-in-research/27372.

50. Braun, V., & Clarke, V. (2021). Thematic Analysis: A Practical Guide. London: Sage

51. Braun V, Clarke V. Using thematic analysis in psychology. Qualitative Research in Psychology. 2006;3(2):77-101.

52. Braun V, Clarke V. Reflecting on reflexive thematic analysis. Qualitative Research in Sport, Exercise and Health. 2019;11(4):589-97.

53. Braun V, Clarke V. Can I use TA? Should I use TA? Should I not use TA? Comparing reflexive thematic analysis and other pattern-based qualitative analytic approaches. Counselling and Psychotherapy Research. 2021;21(1):37-47.

54. Cane J, O'Connor D, Michie S. Validation of the theoretical domains framework for use in behaviour change and implementation research. Implementation Science. 2012;7(1):37.

55. Michie S, van Stralen MM, West R. The behaviour change wheel: A new method for characterising and designing behaviour change interventions. Implementation Science. 2011;6(1):42.

For peer review only

For peer review only

For peer review only - http://bmjopen.bmj.com/site/about/guidelines.xhtml

Figure legend

Figure 1. Study process and activities, mapped to the 4 design thinking phases of empathise (with target population); define (target population's needs, their problems and your insights); ideate (by challenging assumptions and creating ideas for innovative solutions); prototype (to start creating solutions). (44)

For peer review only

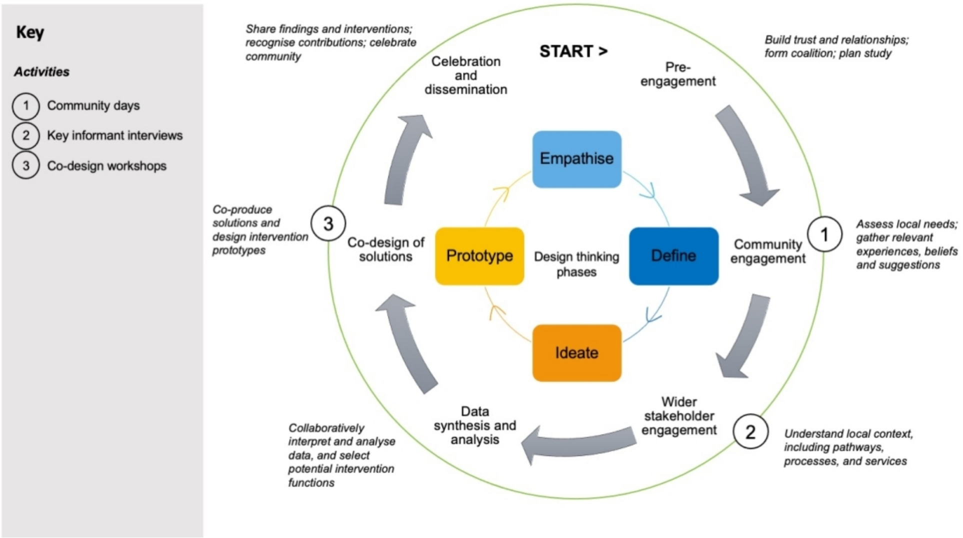

Study process and activities, mapped to the 4 design thinking phases of empathise (with target population); define (target population’s needs, their problems and your insights); ideate (by challenging assumptions and creating ideas for innovative solutions); prototype (to start creating solutions).

338x190mm (118 x 118 DPI)

Completed SRQR Reporting Checklist

[Standards for Reporting Qualitative Research: A Synthesis of Recommendations](#)

O'Brien, Bridget C.; Harris, Ilene B.; Beckman, Thomas J.; Reed, Darcy A.; Cook, David A.

Academic Medicine89(9):1245-1251, September 2014.

doi: 10.1097/ACM.0000000000000388

No.	Topic	Item	Included in study protocol?
	**Title and abstract**		
S1	Title	Concise description of the nature and topic of the study Identifying the study as qualitative or indicating the approach (e.g., ethnography, grounded theory) or data collection methods (e.g., interview, focus group) is recommended	Yes
S2	Abstract	Summary of key elements of the study using the abstract format of the intended publication; typically includes background, purpose, methods, results, and conclusions	Yes
	**Introduction**		
S3	Problem formulation	Description and significance of the problem/phenomenon studied; review of relevant theory and empirical work; problem statement	Yes
S4	Purpose or research question	Purpose of the study and specific objectives or questions	Yes
	**Methods**		
S5	Qualitative approach and research paradigm	Qualitative approach (e.g., ethnography, grounded theory, case study, phenomenology, narrative research) and guiding theory if appropriate; identifying the research paradigm (e.g., postpositivist, constructivist/ interpretivist) is also recommended; rationale[b]	Yes
S6	Researcher characteristics and reflexivity	Researchers' characteristics that may influence the research, including personal attributes, qualifications/experience, relationship with participants, assumptions, and/or presuppositions; potential or actual interaction between researchers' characteristics and the research questions, approach, methods, results, and/or transferability	Yes
S7	Context	Setting/site and salient contextual factors; rationale[b]	Yes
S8	Sampling strategy	How and why research participants, documents, or events were selected; criteria for deciding when no further sampling was necessary (e.g., sampling saturation); rationale[b]	Yes
S9	Ethical issues pertaining to human subjects	Documentation of approval by an appropriate ethics review board and participant consent, or explanation for lack thereof; other confidentiality and data security issues	Yes
S10	Data collection methods	Types of data collected; details of data collection procedures including (as appropriate) start and stop dates of data collection and analysis, iterative process, triangulation of sources/methods, and modification of procedures in response to evolving study findings; rationale[b]	Yes
S11	Data collection instruments and technologies	Description of instruments (e.g., interview guides, questionnaires) and devices (e.g., audio recorders) used for data collection; if/how the instrument(s) changed over the course of the study	Yes
S12	Units of study	Number and relevant characteristics of participants, documents, or events included in the study; level of participation (could be reported in results)	Yes
S13	Data processing	Methods for processing data prior to and during analysis, including transcription, data entry, data management and security, verification of data integrity, data coding, and anonymization/deidentification of excerpts	Yes
S14	Data analysis	Process by which inferences, themes, etc., were identified and developed, including the researchers involved in data analysis; usually references a specific paradigm or approach; rationale [b]	Yes
S15	Techniques to enhance trustworthiness	Techniques to enhance trustworthiness and credibility of data analysis (e.g., member checking, audit trail, triangulation); rationale[b]	Yes
	**Results/findings**		
S16	Synthesis and interpretation	Main findings (e.g., interpretations, inferences, and themes); might include development of a theory or model, or integration with prior research or theory	N/A
S17	Links to empirical data	Evidence (e.g., quotes, field notes, text excerpts, photographs) to substantiate analytic findings	N/A
	**Discussion**		
S18	Integration with prior work, implications, transferability, and contribution(s) to the field	Short summary of main findings; explanation of how findings and conclusions connect to, support, elaborate on, or challenge conclusions of earlier scholarship; discussion of scope of application/ generalizability; identification of unique contribution(s) to scholarship in a discipline or field	N/A
S19	Limitations	Trustworthiness and limitations of findings	Yes
	**Other**		
S20	Conflicts of interest	Potential sources of influence or perceived influence on study conduct and conclusions; how these were managed	Yes
S21	Funding	Sources of funding and other support; role of funders in data collection, interpretation, and reporting	Yes

[a]The authors created the SRQR by searching the literature to identify guidelines, reporting standards, and critical appraisal criteria for qualitative research; reviewing the reference lists of retrieved sources; and contacting experts to gain feedback. The SRQR aims to improve the transparency of all aspects of qualitative research by providing clear standards for reporting qualitative research.
[b]The rationale should briefly discuss the justification for choosing that theory, approach, method, or technique rather than other options available, the assumptions and limitations implicit in those choices, and how those choices influence study conclusions and transferability. As appropriate, the rationale for several items might be discussed together.

ACADEMIC MEDICINE

Standards for Reporting Qualitative Research (SRQR)a

Wolters Kluwer

Copyright © 2022 by the Association of American Medical Colleges
